# A Statistically Rigorous Method for Determining Antigenic Switching Networks

**DOI:** 10.1371/journal.pone.0039335

**Published:** 2012-06-22

**Authors:** Robert Noble, Mario Recker

**Affiliations:** Department of Zoology, University of Oxford, Oxford, United Kingdom; University of Swansea, United Kingdom

## Abstract

Many vector-borne pathogens rely on antigenic variation to prolong infections and increase their likelihood of onward transmission. This immune evasion strategy often involves mutually exclusive switching between members of gene families that encode functionally similar but antigenically different variants during the course of a single infection. Studies of different pathogens have suggested that switching between variant genes is non-random and that genes have intrinsic probabilities of being activated or silenced. These factors could create a hierarchy of gene expression with important implications for both infection dynamics and the acquisition of protective immunity. Inferring complete switching networks from gene transcription data is problematic, however, because of the high dimensionality of the system and uncertainty in the data. Here we present a statistically rigorous method for analysing temporal gene transcription data to reconstruct an underlying switching network. Using artificially generated transcription profiles together with *in vitro var* gene transcript data from two *Plasmodium falciparum* laboratory strains, we show that instead of relying on data from long-term parasite cultures, accuracy can be greatly improved by using transcription time courses of several parasite populations from the same isolate, each starting with different variant distributions. The method further provides explicit indications about the reliability of the resulting networks and can thus be used to test competing hypotheses with regards to the underlying switching pathways. Our results demonstrate that antigenic switch pathways can be determined reliably from short gene transcription profiles assessing multiple time points, even when subject to moderate levels of experimental error. This should yield important new information about switching patterns in antigenically variable organisms and might help to shed light on the molecular basis of antigenic variation.

## Introduction

For pathogenic organisms whose opportunities for transmission are episodic, rather than continuous, long infectious periods are crucial for successful transmission. Most notably vector-borne and sexually transmitted pathogens have evolved various strategies to increase their transmission potential by evading their hosts’ immune responses. One of the most sophisticated strategies is antigenic variation whereby the pathogen repeatedly changes its antigens over the course of a single infection. Although the underlying mechanisms differ between pathogens, many involve a polymorphic family of genes encoding functionally similar but antigenically diverse variants (reviewed in [1]). Of these genes, only one is actively transcribed while all other genes are transcriptionally silent. During infection, the active gene can ‘switch off’, leading to the activation, or ‘switching on’, of a previously silent gene. It is believed that this mono-allelic gene expression pattern helps the pathogen to guard its available antigenic repertoire from the immune system. Furthermore, gene expression has to be coordinated across the whole parasite population, such that the majority of the population expresses the same gene at the same time, as the host would otherwise build up immunity to all variants early on and clear the infection prematurely. As a result, infections with antigenically variable pathogens are often characterised by successive waves of parasitemia that are sequentially dominated by one or only a few antigenic variants.

Some of the best studied organisms employing antigenic variation are African trypanosomes and the causative agent of severe malaria in humans *Plasmodium falciparum*. Various theoretical studies have concentrated on determining the underlying mechanisms responsible for the observed coordination in antigen presentation during infection by these pathogens. In particular, gene activation hierarchies or differences in growth rates have been put forward as potential drivers behind their characteristic infection dynamics [2]–[7]. Although it was found that parasite intrinsic factors could orchestrate the parasite population in the initial phases of infection, they are insufficient for maintaining sequential dominance of antigenic variants during the later, chronic stages of infections. Instead, immune selection via cross-reactive immune responses has been proposed to offer a more parsimonious solution to this problem, even in the absence of structured differences in switch or growth rates [8]. Nevertheless, *in vitro* studies of malaria parasites have since shown that variant switching is non-random and partly gene specific. For example, Horrocks *et al.* [9] demonstrated that *var* genes, which encode the surface-expressed virulence factor PfEMP1 (*P. falciparum* erythrocyte membrane protein 1), switch on and off at different but constant rates during long term culture. Frank *et al.* [10] also found that these rates differ widely between different genes and that centrally located genes appear to have lower switch rates than those in subtelomeric loci. This has recently been confirmed by Enderes *et al.* [11] who also showed that these switch preferences appear to be independent of genetic background. Similarly, in *Trypanosoma brucei* it has been shown that switching between *vsg* genes, which encode the pathogens surface coat, is determined by a fixed hierarchy of activation probabilities that appear to depend on features of the genes’ loci [7], [12], [13]. An expression hierarchy dependent on two particular extragenic elements has also been found to underlie antigenic variation in the spirochete *Borrelia hermsii* [14]. Such observations have led to the hypothesis that structured switching or switch hierarchies might be important for structuring the parasite population during the early stages of infection. On the one hand this would make it easier for the adaptive immune response to desynchronise the parasite population and set up a pattern whereby single variants can successively dominate the infection [8]. On the other hand it would also help the parasite to establish an infection in individuals with pre-existing immune responses, as recently shown in [15].

To distinguish between parasite-intrinsic switching and host mediated selection during infection it is important to have a thorough understanding of inherent switch patterns. One approach to investigate these patterns is to analyse longitudinal gene transcription data from *in vitro* cultured parasites in the absence of selection. Given sufficient transcription data, it should theoretically be possible to determine the switching network of a complete gene repertoire. However, a major challenge is the large sample space which makes simple methods unreliable or impractical. For instance, to fully determine the switching network of a pathogen with a repertoire of 10 variant genes requires 90 parameters to describe every gene’s rate of activation and silencing (assuming that each gene’s activation probability may depend on which gene is being switched off). Using a grid search method testing five values for each parameter would require 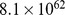 simulations and would probably miss the globally optimal solution by a wide margin. Recently, Recker *et al.* [15] addressed this problem using an iterative algorithm to determine the switching pattern of *P. falciparum var* genes, using data from clonal parasite populations followed over 60–80 generations, with *var* transcription levels measured at regular intervals using quantitative ‘real-time’ PCR (qrt-PCR). Although this method could robustly determine major switch pathways, it was able to explore only a small subset of the many-dimensional parameter space. Furthermore, qrt-PCR data are subject to measurement error, especially when transcription levels are very low, and the algorithm could not provide an indication of the uncertainty of its parameter estimates.

Here we present a statistically rigorous solution to this problem in the form of simulated annealing and Markov Chain Monte Carlo (MCMC) algorithms. Using diverse sets of artificially generated gene transcription time courses we demonstrate that highly structured and complex switch pathways can be resolved reliably from relatively limited data. We further show that although experimental noise can have a major effect on estimates, using transcription measurements from several populations of the same pathogen strain can significantly improve accuracy. Our method can thus be used to resolve complex and high-dimensional switch patterns with high reliability and accuracy from limited and noisy data.

## Results

The general aim of our method is to find a set of parameter values relating to antigenic switching, i.e. the switch rates and activation biases, that best fit temporal variant transcript distributions. Under the assumption that a variant’s switch rate and biases are constant over time we can describe the proportion of a parasite population transcribing gene variant *i* at time 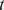, 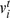, simply as
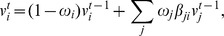
where 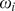 is the rate at which gene 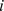 is switched off and 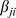 is the switch bias, or probability of a switch from variant 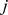 to variant 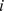. The switch network or pathway underlying a measured change in transcription levels can thus be described by the combination of a switch matrix 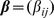 and off-rate vector 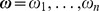. The task then is to use an iterative approach (see Methods) to find 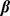 and 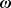 such that the deviation between the measured and simulated transcript levels at time points 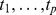 is minimised.

### Method Testing

To test our method for accuracy and reliability in resolving antigenic switch patterns from gene transcription time courses we used various test parameter sets representing a wide spectrum of possible switch pathways (see Methods). That is, we constructed a number of artificial switch networks of different degrees of complexity, in terms of the number of genes that each gene switches to, and the number of consecutive switch events, and used these to generate temporal gene transcription data. We then applied our methods in order to reconstruct the most likely switch pathway underlying the data.

In line with previous *var* gene transcription studies we assumed that the parasite population is initially clonal, with every parasite transcribing the same gene, and that relative transcript levels are measured at several time points during *in vitro* culture. A common feature of the previously described transcription profiles is that only a fraction of transcripts reach significant levels during *in vitro* culture [9]–[11], [15]. We have previously argued that less transcribed genes are unlikely to play a dominant role in the switching network in the neighbourhood of the starting gene. We therefore assumed that removing data for genes that are generally transcribed at very low levels would have little effect on the parameter estimates for the interactions between more dominant genes, and that these interactions would be sufficient to determine the most important properties of the switching network. Accordingly, our initial analysis was restricted to a set of 10–16 genes, which could represent the subset of the most dominantly transcribed genes. We later provide a numerical justification for using such a reduced system to determine major transcription pathways.

#### Resolving switch networks from a single transcription time series

**Figure 1:**
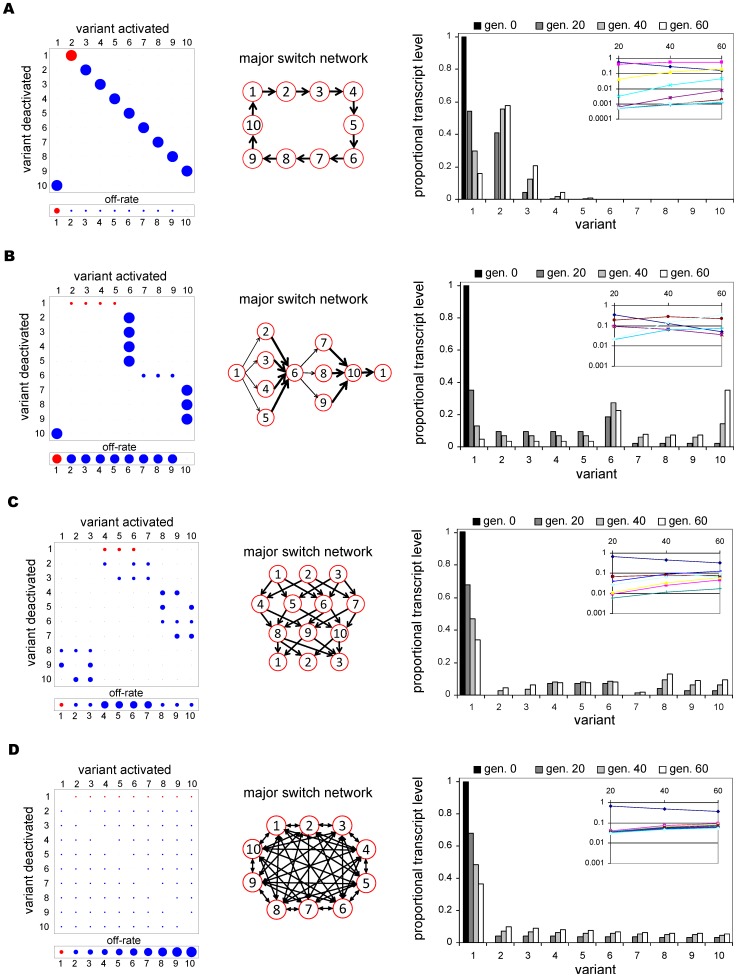
Target network and transcript data for initial testing. Four switch networks of different complexities were considered for the initial testing and method calibration: (**A**) one-to-one (1∶1), (**B**) single-many-single (SMS), (**C**) lattice, and (**D**) uniform. The sizes of the discs in the switch matrices correspond to the transition biases from variant 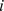 to variant 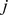, 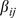, and the sizes of the discs in the off-rate vectors are proportional to the per-generation de-activation rates, 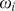. The major switch pathways described by these matrices are highlighted in the middle column and the right column shows the proportional transcription levels of all 10 variants from point of cloning until 60 generations post cloning, taken at 20 generation intervals; the insets depict the proportional transcript levels of the 10 variants on a log scale at generations 20, 40 and 60, with each colour representing a different variant.

Initially we considered four switching networks of different complexities consisting of 10 genes. The first network describes a situation in which each gene simply switches to only one other gene with very high bias, and is referred to as a one-to-one (1∶1) pathway. The second network, referred to as single-many-single (SMS), is similar to that proposed to underlie *var* gene transcription data in a previous study [15]. It describes switching from the initial gene to a group of genes, which all switch with high bias to another single gene. The latter gene in turn switches to a different group of genes, which switch back to the first gene. The third network has a lattice-like structure containing block-diagonal switch biases, and the fourth is a uniform network, where each gene has identical switch biases. The four networks are illustrated in Figure 1 together with the matrix and vector representation of switch biases and off-rates, 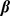 and 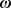 respectively, and the simulated transcription time courses resulting from these networks of up to 60 generations post cloning.

**Figure 2:**
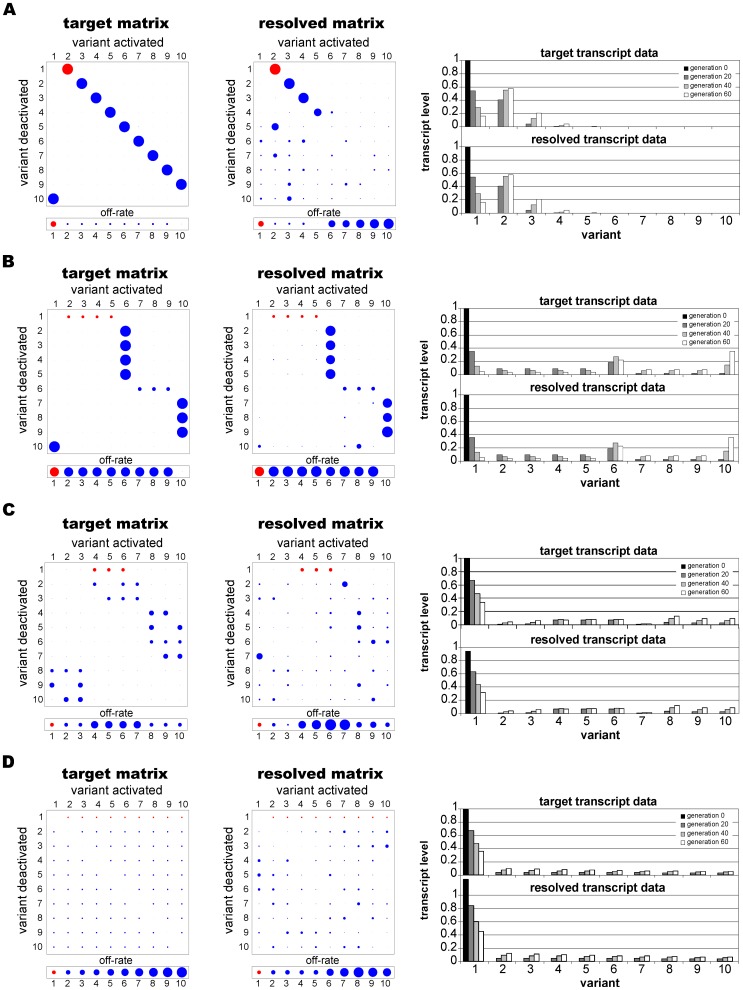
Resolved networks from a single time series. Using simulated annealing the (**A**) 1∶1, (**B**) SMS and (**D**) uniform networks are resolved to up to four consecutive switches, although the complex lattice network (**C**) is less well described. The starter genes, which were assumed to be clonally transcribed at generation 0, are highlighted in red. The simulated time courses (right column) show a very good agreement between the target data and the transcription data resulting from the determined network.

Using simulated annealing (see Methods) we aimed to recover the original switching parameters from the transcript levels measured at generations 20, 40 and 60. Figure 2 shows that in each case the method found a good fit between the target and simulated transcript distributions, and it reliably recovered the first four consecutive switch events of the 1∶1, SMS and uniform target networks. The switch bias matrix and off-rate vector were less well resolved for the later stages of the 1∶1 network (Figure 2A), which comprises ten consecutive switches, and for the lattice network, which has an especially complex structure. Note that parameters associated with the later network stages, i.e. of those genes that are activated only after several switch events, have relatively minor effects on transcript levels and the simulated annealing algorithm is less sensitive to their variation. That is, even when the model output fits the transcript time courses of these variants very tightly (for all networks the average overall error, or deviation between the input data and the model output, was less than 0.1%), the particular pathways between rarely transcribed genes are less accurately described.

**Figure 3:**
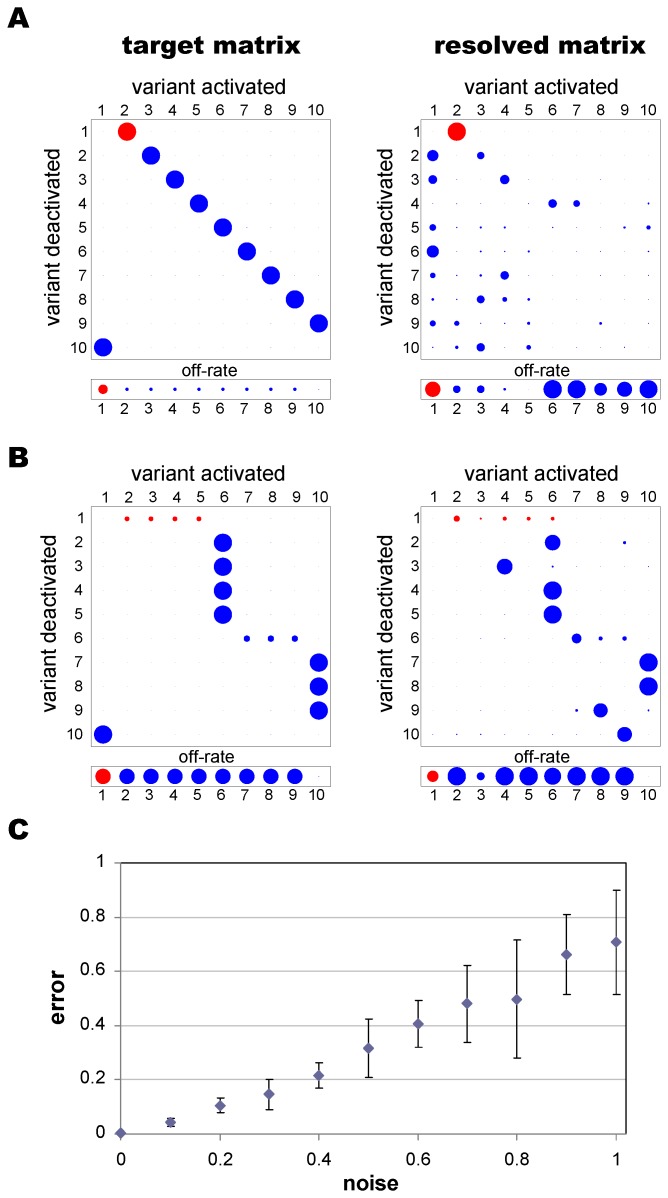
Resolved networks from noisy data. The accuracy in determining switch networks from single transcription time courses is significantly affected by the level of noise in the data (here 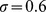) and can lead to poorly resolved networks, as shown for both the (**A**) 1∶1 and (**B**) SMS pathways. (**C**) The error shows a linear correlation with the level of noise.

We next investigated the effect of noise, in terms of experimental error (see Methods), on the method’s ability to recover underlying switch patterns. As shown in Figure 3 and S1, accuracy dropped markedly when noise was added to the data. However, under moderate levels of noise, major switch pathways could still be determined with reasonable accuracy, dependent on the particular network structure. The mean error increased linearly with the noise level (Figure 3C) but there was only a weak correlation between these two variables. It would therefore be impossible to use this error to estimate precisely the degree of noise in an experimental data set. Furthermore, we found a very weak correlation between the noise level and the error in the switch bias estimates (results not shown), indicating that there would be wide variation in the accuracy of parameter estimates derived from experimental data of this type.

To improve the method’s accuracy we increased the number of time points at which transcript levels were measured. For the 1∶1 network with noise added, changing from three to seven time points resulted in a 22% decrease in the root mean square deviation between the output switch biases and the target parameters for the first four network stages. More than doubling the number of measurements to 15 yielded only a 16% additional improvement. This non-linear relationship between the number of time points and the deviation of the parameter estimates indicates that the method’s accuracy in describing the entire switch pathways of a parasite’s gene repertoire is not simply limited by the frequency of measurements.

#### Resolving switch networks from multiple transcription time series

Since our method resolved the underlying switch networks with limited resolution for those genes whose transcript levels remained low over the whole time course, we next tested whether accuracy could be improved by using several target data sets generated by the same network. That is, using the same global switch pathway we generated transcription time courses from different clonal starter populations and then tried to find the most likely switch pathway by fitting the model to all of the data simultaneously. We increased the size of the parasite’s repertoire to consist of 16 genes (requiring 240 parameters) and randomly selected eight of these to be the clonally expressed starter genes for separate data sets, corresponding to eight clones taken from the same parent culture. Transcription levels were recorded for each data set at five time points at generations 20, 30, 40, 60 and 80 post cloning.

**Figure 4:**
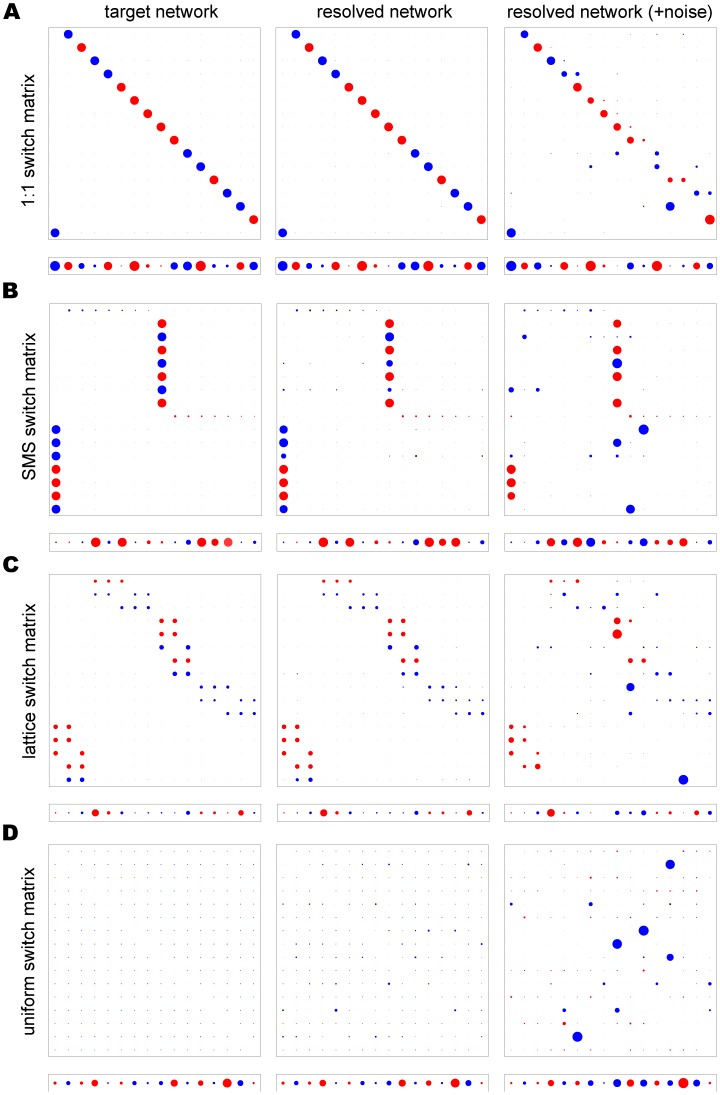
Resolved networks from multiple time series. Transcription histories from eight different clones, each defined by a different starting gene (highlighted in red), were used to resolve four different switch pathways describing 1∶1, SMS, lattice and uniform networks (**A–D**, respectively). Without any noise, all matrices and off-rate vectors can be resolved to a high degree of accuracy, even for the non-starter genes (middle column). The use of multiple data sets also yields better estimates when significant levels of noise are added to the data (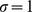, right column).

**Figure 5:**
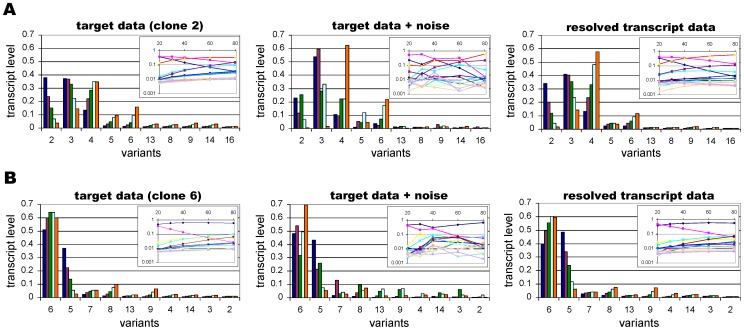
Resolved transcription time courses from noisy data. When the model is fitted to multiple data sets with different starting genes, the transcript levels of the predicted network (right column) are more similar to the noiseless data of the underlying network (left column) than to the noisy data used as input (middle column). Results are shown for two different clones of a 1∶1 switch network with transcript levels measured at generation 20 (blue), 30 (purple), 40 (green), 60 (light blue) and 80 (orange). The insets depict the proportional transcript levels of the 16 variants on a log scale at generations 20, 30, 40, 60 and 80, with each colour representing a different variant.

As shown in Figure 4 (middle column), despite the high complexity of the underlying networks and size of the parameter space all four switch pathways were consistently recovered with high accuracy and resolution by using multiple time series simultaneously. Even when we considered a high degree of experimental noise, the switch bias and off-rates were accurately predicted for the starter genes (highlighted in red). It should be noted that the level of noise considered here was considerably higher than what we would expect of experimental data and corresponds to transcription level measurements typically differing from the true values by a factor of two. Although the parameter estimates for non-starter genes (blue discs) were less reliable at moderate and high noise levels, they were considerably more accurate than would be expected by chance. Switch biases of non-starter genes were recovered most accurately for the 1∶1 network and less accurately for the SMS and lattice network. This is not surprising, however, given the high degree of complexity of these particular switching networks. Furthermore, the noisy data were fitted more closely by the model predictions than by the true, i.e. noise-less, transcription levels (the error was on average 15% smaller, independent of network type or noise level), which confirms that inaccuracies in the parameter estimates were mostly due to the noise and not to a failure of our method to locate good optima. Figure 5 shows two examples of the transcription time courses of the 10 most highly transcribed genes from two different starter genes (2 and 6) of an underlying 1∶1 switch pathway, with and without added noise, together with the transcript distributions resulting from the estimated switching networks. This again highlights the robustness of this method to determine genetic switch patterns from limited and noisy data.

Similarly to when we used just one transcript time series, the error increased linearly with the noise level but this time the two variables were strongly correlated (data not shown). The error value obtained from a sufficiently large set of experimental data would therefore be a good estimator of the degree of experimental noise in the data and could be used as an input parameter for determining the uncertainty of the resolved switching network using a Markov Chain Monte Carlo (MCMC) approach described below.

#### MCMC

One explanation for the inaccuracy of the simulated annealing method in estimating the switch parameters for non-starter genes, and generally genes that remain at low transcript levels during the whole experiment, is that the error is less sensitive to their variation. This would imply the existence of alternative switch networks that fit the target data almost as well as the predicted best-fit solution. To investigate the level of uncertainty and obtain a probability distribution for each parameter, we used a Markov Chain Monte Carlo (MCMC) method (see Methods).

**Figure 6:**
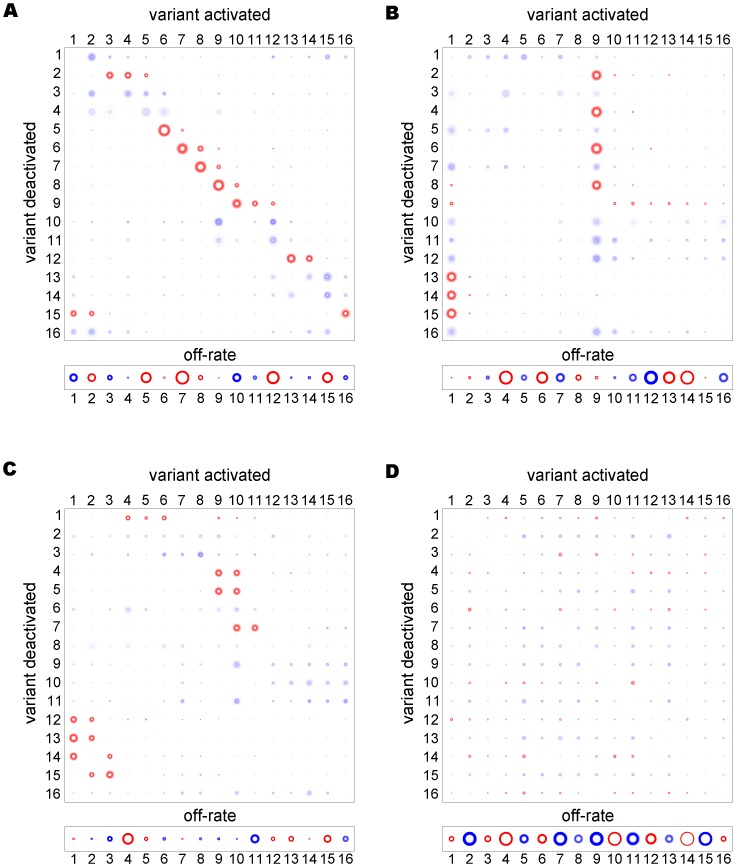
Using MCMC on noisy transcription data. In the MCMC output for the four different switch pathways: 1∶1, SMS, lattice and uniform, (**A–D**, respectively), the parameter range for each switch bias and off-rate was divided into bins, represented by rings. If a large proportion of recorded solutions contained similar values for a parameter then the corresponding ring is coloured dark, indicating a high likelihood that the true parameter value lies within that range. The proportions were measured relative to a null distribution 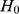, which assumed that all solutions were equally likely to be accepted. The darkest colour corresponds to proportions that differed from the 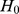 mean by at least 25 standard deviations.

As demonstrated in Figure 6, using the same noisy transcription time series as in Figure 4, we found that the MCMC method produced generally accurate and precise estimates of starter gene parameters (indicated by less fuzzy rings) in all four network types. Moreover, off-rates for non-starter genes were estimated with a good degree of accuracy and precision, except those for the uniform network which were consistently overestimated. Although estimates of the switch rates of non-starter genes were less precise they were still informative. As is evident from comparing the MCMC output in Figure 6 to the output from simulated annealing (right column of Figure 4), the MCMC method provides a much more reliable indication of the true switching structure. However, the simulated annealing algorithm is still required to estimate the level of noise in the data used to calibrate the MCMC method.

#### Dimension reduction

We initially argued that using data from only the most dominant genes within the repertoire is justifiable as long as the majority of gene transcripts remain at very low levels throughout the experiment. To verify this assumption we created 60-dimensional versions of our four test networks. Starting genes were selected by first simulating transcription histories for many generations until the transcript levels were close to equilibrium and thus resembled a parent population kept in long-term culture. Eight distinct starting genes were then randomly selected, such that the probability of selecting each gene was proportional to its level of transcription in the parent culture, thus mimicking experimental selection of starting genes by limiting dilution.

**Figure 7:**
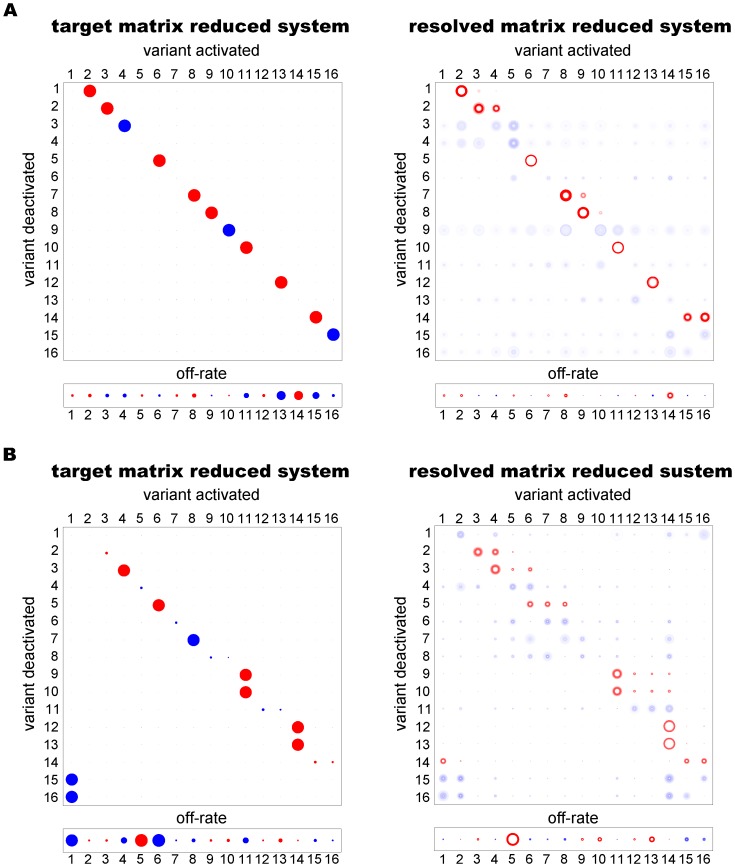
Results following dimension reduction. Left column: target parameters for the (**A**) 1∶1 and (**B**) SMS networks reduced from 60 to 16 genes. Right column: MCMC output after adding noise with 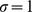. To perform the reduction, genes were ranked by their average transcription levels across all time points and all cultures in the data generated by the 60-dimensional matrix (after adding noise). The 16 most highly ranked genes were then selected and their data renormalised. The starter gene parameters are shown in red.

For each network we applied the MCMC algorithm using transcription data for only the 16 most transcribed genes. To perform the reduction, genes were ranked by their average transcription levels across all time points and all cultures in the data generated by the 60-dimensional matrix (after adding noise). Data for the 16 most highly ranked genes were then selected, renormalised, and used as input. Note that genes chosen in this way may come from diverse regions of the switching network, so that the reduced matrix, which represents disjoint network elements, may look different from the original, full matrix. For example, the reduced SMS and 1∶1 matrices (Figure 7A and 7B) are somewhat similar. Later we show how hypothesis testing may be used to determine the most likely network type, even when the MCMC output appears ambiguous.

For each of our reduced test networks, the MCMC parameter estimates gave a good indication of the network structure (Figure 7 and S2). Where estimates varied from the true parameter values, this was often because genes included in the reduced system took on roles in the network vacated by omitted genes, so that the general network structure was preserved. These results confirm that accurate estimates of the overall switch pathways can be obtained by using only a subset of highly transcribed genes.

### Application

As demonstrated in the previous section, our method can reliably determine genetic switch pathways from relatively limited data; however, it also showed how parameter estimates can be affected by experimental noise or sparseness of the available data to be fitted. And although the MCMC approach provides a good indication of the uncertainty in parameter values and thus the underlying switching pathway, the question remains how to proceed if the method results in ambiguous outputs. Here we provide one approach in terms of hypothesis testing, which allows the direct comparison of the likelihoods of alternative switch pathways. Finally we apply our method to real *var* gene transcription data previously described in [15].

#### Hypothesis testing

A strength of our likelihood-based approach is that it allows testing of hypotheses regarding the network structure where the predicted switch pathway is not immediately obvious or where there is a high degree of uncertainty around the estimated parameter values comprising this pathway. That is, if the outcome of the MCMC method is ambiguous in terms of the most likely switching network underlying the observed change in transcription levels, we can use this approach to specifically test different hypotheses. To demonstrate how this technique might be useful in practice we used likelihood ratio tests to compare the power of two alternative switching models to explain the transcription data generated by the networks considered in the previous section (1∶1, SMS, lattice and uniform networks). In the first model, all genes were assumed to have identical, uniform switch biases, corresponding to a situation with completely unbiased switching; the second model allowed all genes to have different sets of switch biases, as in the structured switch pathways considered previously. We then used simulated annealing to find the maximum likelihoods of each model using the noisy, dimension-reduced data sets for each network.

For the 1∶1, SMS and lattice networks, the likelihood ratios correctly indicated that, in each case, the switch biases were unlikely to be uniform (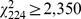, 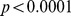), implying local switch biases and thus rejecting the hypothesis that a change in transcript levels would simply be due to differences in off-rates. Conversely, for the uniform network the test outcome was consistent with uniform switch biases (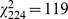, 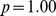), thus providing no support for the notion of variant-specific switch biases in this case. It is also possible to test for differences between the parameters of specific genes. For example, likelihood ratio tests indicated that in the SMS matrix, the switch biases of gene 10 were likely to differ from those of gene 12 (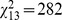, 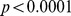) but not from those of gene 9 (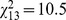, 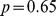). The latter result implies, correctly, that the network was unlikely to have a 1∶1 structure.

Results of F-tests were similar to those of the likelihood ratio tests, and other model selection statistics such as Bayes factors or information criteria are also compatible with our method. Thus, given sufficient experimental data, it should be possible to determine with a high degree of confidence whether a hypothesised switching pattern is likely to be correct.

#### Experimental data

**Figure 8:**
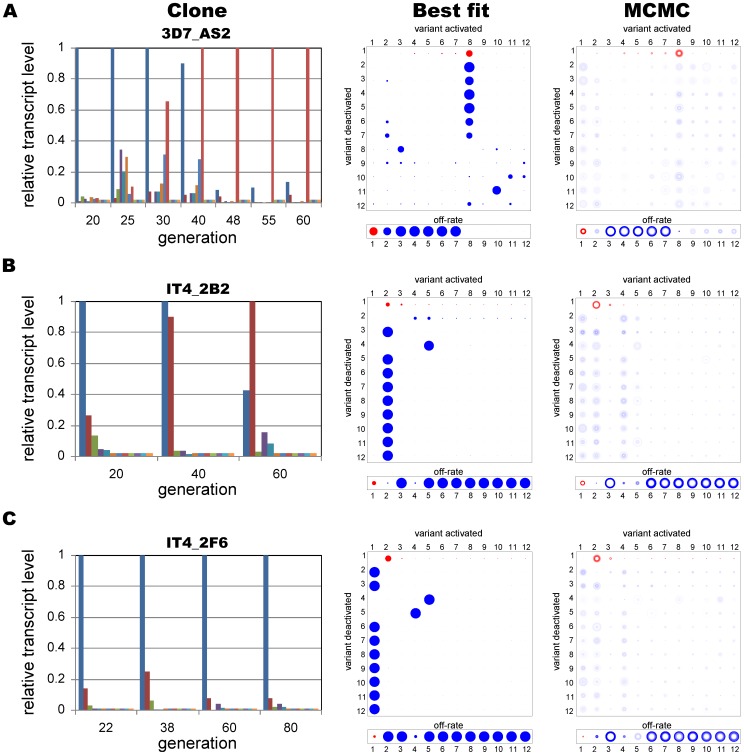
Results from experimental data. Best-fit parameter estimates derived by simulated annealing (top row) and MCMC parameter distributions (bottom row) are shown for three sets of *P. falciparum var* gene transcription data previously analysed by Recker *et al.* [15]. Each data set comprises a single time series of measurements from an initially clonal culture. The results are consistent with an SMS network structure although the MCMC output (right column) also indicates the likelihood of alternative pathways.

Finally, we applied our methods to experimental data, comprising a subset of the *P. falciparum var* gene transcription measurements of three different clones (3D7_AS2, IT4_2B2 and IT4_2F6) previously analysed by Recker *et al.* [15]. In agreement with the proposed switch pathways, for each data set the simulated annealing algorithm found that an SMS (single-many-single) network structure gave the best fit to the data. Because of the sparsity of those particular data sets we used the MCMC-based method to assess the uncertainty in the parameter estimates. Although the results confirmed that the switching network was unlikely to have a 1∶1 or uniform structure, the output also revealed that other parameter sets representing different network structures were equally likely, as indicated by the blurry matrix entries in Figure 8; unfortunately the data were insufficient to carry out the more detailed hypothesis testing outlined above. Note, we obtained similar results when we used a single, artificial data set generated by a SMS matrix (Figure S3), which strongly indicates that more data are required for determining the precise pattern of antigenic switching among *var* genes.

## Discussion

Here we described a statistically rigorous method for determining genetic switch pathways from quantitative gene transcription data. The problem in determining switch pathways with direct methods is the wealth of data required to estimate gene intrinsic parameters, such as activation biases and off-rates, especially when the system under consideration consists of more than just a couple of genes. Most antigenically variable organisms have gene repertoires that are orders of magnitude larger, however. We have demonstrated that our iterative method is able to resolve complex switch patterns from minimal data to a high degree of accuracy, even when allowing for a reasonable degree of experimental error or noise. Importantly, we have shown that using multiple sets of data simultaneously increases accuracy such that even highly complex networks can be determined. This should be of particular interest as it suggests that the most reliable information about a gene’s or gene repertoire’s switch behaviour can be extracted not necessarily from long-term cultures but from analysing transcription profiles which are relatively short but obtained from different clones of the same isolate. In general, our results suggest the following strategy for determining genetic switching networks in three stages. The first step would be to estimate the amount of noise in the data, using simulated annealing. This would be followed by employing our MCMC method – calibrated to the estimated noise level – to return reliable parameter distributions for the parameters comprising the underlying network, i.e. the genes’ off-rates and switch biases. Lastly, and strongly dependent on the amount and quality of the available data, one would test hypothesised network structures by comparing the likelihoods of restricted models.

For structured networks we noticed that some variant transcripts remain at very low levels over the whole transcription time courses considered here. This is consistent with experimental studies of longitudinal *var* gene transcription, which also found that some variants are rarely activated, even after long-term *in vitro* culture. Although we could show that data for only the most dominantly transcribed genes are sufficient for determining the overall switch pattern, uncertainty in parameter estimates for genes with low activation probabilities remains a problem for describing the switch behaviour of an entire gene repertoire. However, once identified it should be possible to specifically select these genes for cloning and then apply the method described here. Knowledge about genes with low activation probabilities *in vitro* could yield important information about *in vivo* selection processes and should help to distinguish between parasite-intrinsic switching and immune- or receptor-mediated selection underlying the within-host infection dynamics of antigenically variable organisms.

Investigating intrinsic switch patterns is of major importance for a number of reasons. First of all, the non-random nature at which genes are activated or silenced is likely the result of an evolutionary process shaped by the interaction between the parasite and the immune system. Understanding this pattern would therefore provide valuable insights into the selective pressure acting upon the parasite during infections, which in turn should also shed light on the evolution and structure of the gene repertoire itself. For *P. falciparum* at least, there also is the added complexity of phenotypic as well as antigenic variation. Different PfEMP1 variants mediate cytoadherence of infected red blood cells to different host tissues [16], [17] and expression of certain subsets of the *var* repertoire has been associated with disease severity and young host age [18]–[20]. A full characterisation of the antigenic repertoire in terms of gene activation rates and hierarchies will therefore help to explain age-related pathologies of malaria infections and the observed order of acquisition of protective immunity against certain subsets of genes. It is also hoped that analysing pre-determined switch patterns will contribute to our understanding of the molecular mechanisms behind antigenic variation. For example, differentiation of switch biases between particular genes and “universal” activation preferences should help elucidate how antigenic switching is controlled at the genetic and/or epigenetic level.

Although our method was described with reference to a particular *in vitro* experimental set-up in which *P. falciparum* parasites are followed from an initially clonal population over time, there is no restriction on how the data is generated. In fact, provided that adequate temporal gene transcription data can be obtained, either from *in vitro* culture or *in vivo* infection dynamics, this method can be used to reliably determine the parameters that define the switching network between genes in any antigenically variable organism.

### Methods

To analyse antigenic switch pathways from gene transcription data we follow the approach taken by Recker *et al.* [15]. We assume that clonal parasite populations are followed over an extended period of *in vitro* culture with relative transcript levels of all genes measured at various time points. The resulting transcription profiles can then be described by the following time-discrete model:
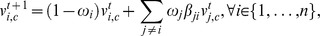
where 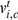 is the relative transcript level of variant 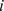 in culture 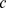 at time 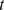, 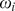 is the variant specific off-rate, and 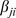 is the probability of a switch from variant 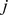 to variant 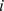. The task is then to find the parameter values for 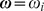 and 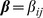 that minimise an error function defined by the deviation between the measured – or in our case simulated – transcript level 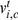 and the model outcome 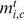:



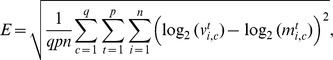
where 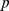 is the number of time points where transcript levels were measured and 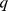 is the number of cultures.

We assumed that off-rates do not exceed 6% per generation, which is consistent with experimental measurements [9], [10], [12]–[14], [21].

### Simulated Annealing

Because of the large dimension of the system we used iterative approaches to find solutions that minimised the deviation between the model output and the data. Simulated annealing is a probabilistic optimisation algorithm which can move between locally optimal parameter sets [22]. The procedure starts with a random parameter set 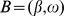 containing the switch biases and off-rates, and applies a random perturbation to produce 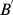. The move from 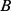 to 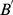 is accepted if it reduces the error; otherwise the move is accepted with a probability dependent on how much it increases the error and on a “temperature” parameter. The perturbation and acceptance steps are repeated for many iterations while the temperature is gradually lowered so that moves that increase the error become less likely to be accepted. When the temperature becomes sufficiently low the parameter set converges to a local minimum.

Perturbation of a switch bias parameter 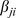 was achieved by drawing a random variable 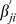 from a Cauchy distribution centred at 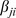. The distribution was restricted to the permitted range [0,1] by mapping
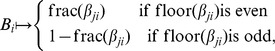
where floor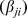 is the largest integer not greater than 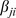 and frac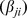 is the fractional part. Off-rates were similarly perturbed after rescaling in proportion to the parameter range [0,0.06]. In each iteration the perturbation procedure was applied to one switch bias per matrix row and the other entries were then rescaled to normalise the row. All off-rates were perturbed in each iteration. We chose a Cauchy distribution because it has a fatter tail than the Gaussian distribution and may therefore be more likely to generate “basin hopping” moves between local optima.

This perturbation procedure is symmetrical, meaning that the probability of moving from 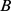 to 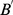 is always equal to the probability of moving from 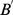 to 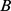. However, normalising each row of switch biases caused the algorithm disproportionately to favour small parameter values. Such non-uniformity is permitted in simulated annealing and should not affect the results. The perturbation size was determined by the scale parameter of the Cauchy distribution, which was made constant.

For the acceptance threshold we chose the Fermi distribution; that is, proposed moves were accepted with probability 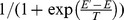, where 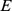 and 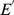 are the errors associated with the current and proposed parameter sets respectively, and 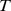 is the temperature. We used a geometric cooling schedule and stopped the algorithm after 10 million iterations.

### Experimental Error

Gene transcript levels are calculated from qrt-PCR output using an exponential formula in which the base is the amplification efficiency of the gene transcript (which should be approximately 2) and the exponent is the number of amplification cycles required for the transcript to reach a threshold abundance [23]. We therefore assumed that error-prone measurements would follow a log-normal distribution and that, at least for the most abundant transcripts, the standard deviation would be proportional to the mean. Accordingly, measurement errors were simulated by applying a noise function 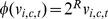, with 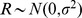 to the simulated transcription profiles and then renormalising each set 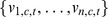. In most of our trials 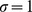, which assumes that transcript levels are typically measured to be two times larger or smaller than their true values.

### MCMC

To obtain distributions of likely values for the switch biases and off-rates, instead of only the “best-fit” point estimates, we used a Markov chain Monte Carlo (MCMC) method known as the Metropolis-Hastings algorithm [24], [25]. This algorithm accepts proposed moves with probability 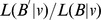, which is the likelihood ratio of the proposed parameter set 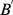 and the current parameter set 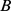, given the observed data 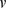. To convert the simulated annealing algorithm to instead perform MCMC we replaced the acceptance threshold with the exact likelihood ratio, as derived below, and used the simulated annealing error to estimate the noise parameter 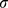. The algorithm was run for 10 million iterations and every accepted solution was recorded after an initial “burn-in” period of 1 million iterations.

To simplify the notation, the following derivation considers the case with only one culture and one time point, so 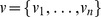 is the observed data and 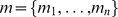 is the output of the model with parameter set 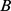. Assuming as before that each 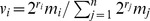, where each 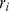 is an instance of a random variable 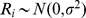, then
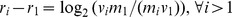
and



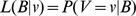


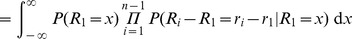


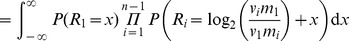


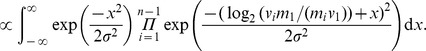
Changing the variable to 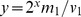 gives



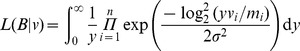


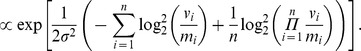
It follows that the likelihood ratio for 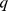 cultures and 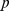 time points is



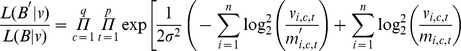


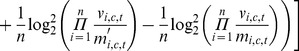


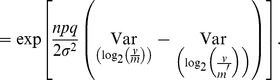



## Supporting Information

Figure S1
**Resolved networks from noisy data.** The accuracy in determining switch networks from single transcription time courses is significantly affected by the level of noise in the data (here 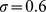) and can lead to poorly resolved networks, as shown for both the (**A**) lattice and (**B**) uniform pathways.(TIFF)

Figure S2
**Results following dimension reduction.** Left column: target parameters for the (**A**) lattice and (**B**) uniform networks reduced from 60 to 16 genes. Right column: MCMC output after adding noise with 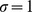. To perform the reduction, genes were ranked by their average transcription levels across all time points and all cultures in the data generated by the 60-dimensional matrix (after adding noise). The 16 most highly ranked genes were then selected and their data renormalised. The starter gene parameters are shown in red.(TIFF)

Figure S3 **Results from simulating hypothesised** ***var*** **gene system.** In this case the 60-gene SMS network was reduced to 12 genes by selecting only one starter gene, instead of the eight starter genes used in Figure 7. Simulated transcription levels were recorded at only three time points and a high level of noise was added to the data. These conditions mimic those of the experimental data sets IT4_2B2 (three time points recorded) and IT4_2F6 (four time points) analysed by Recker *et al.* [15] and the results are similar to those found by analysing the original data (Figure 8). The best-fit parameter estimates suggest an SMS network but the MCMC output shows that this result is uncertain because of the relatively small data set.
(TIFF)
