# Supplementary figures and images for: A Statistically Rigorous Method for Determining Antigenic Switching Networks
Source: PLoS One. 2012 Jun 22;7(6):e39335. doi: 10.1371/journal.pone.0039335 (PMC3382232; doi:10.1371/journal.pone.0039335)

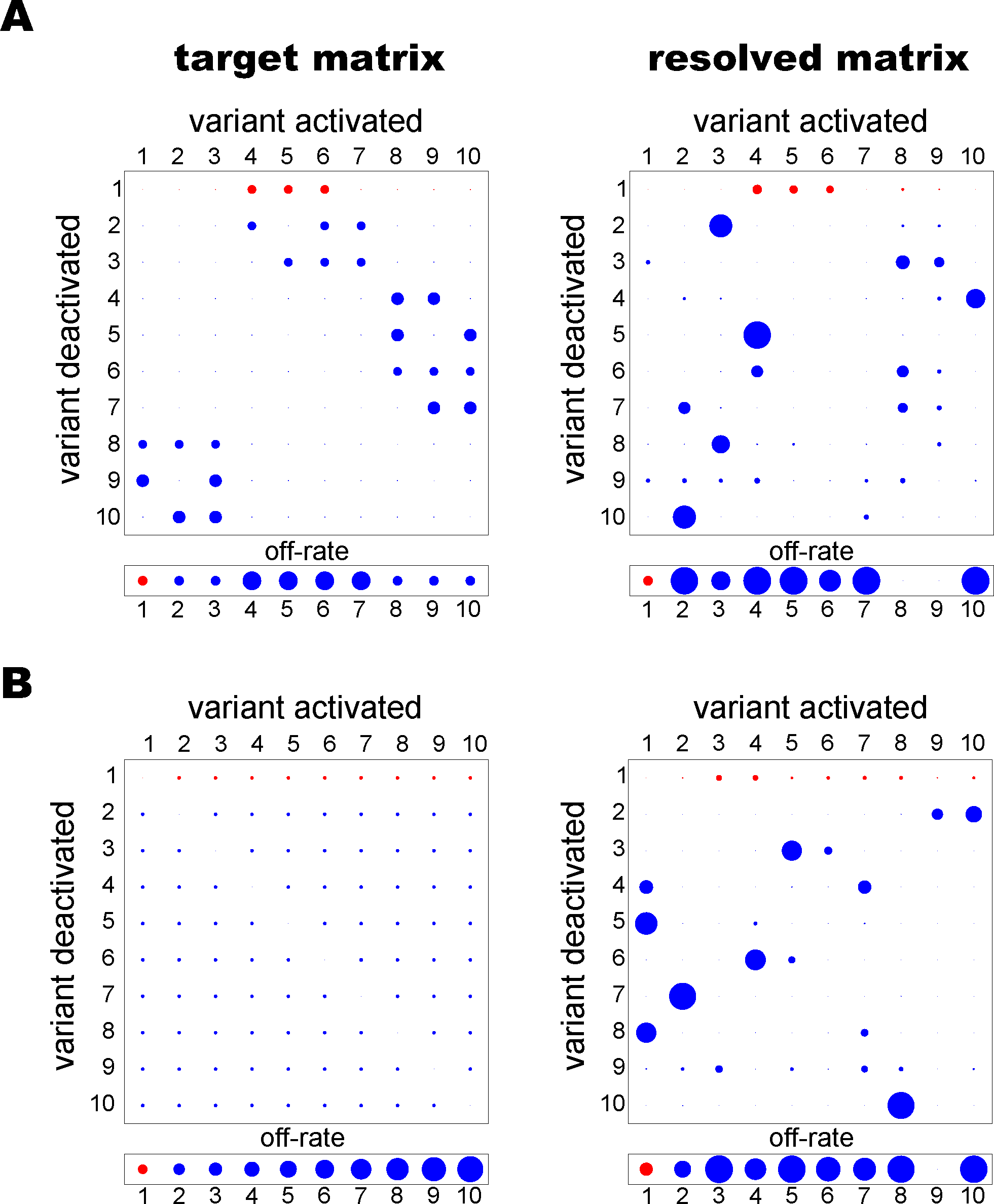

Supplement: Figure S1 — Resolved networks from noisy data. The accuracy in determining switch networks from single transcription time courses is significantly affected by the level of noise in the data (here ) and can lead to poorly resolved networks, as shown for both the (A) lattice and (B) uniform pathways. (TIFF) [file pone.0039335.s001.tiff]

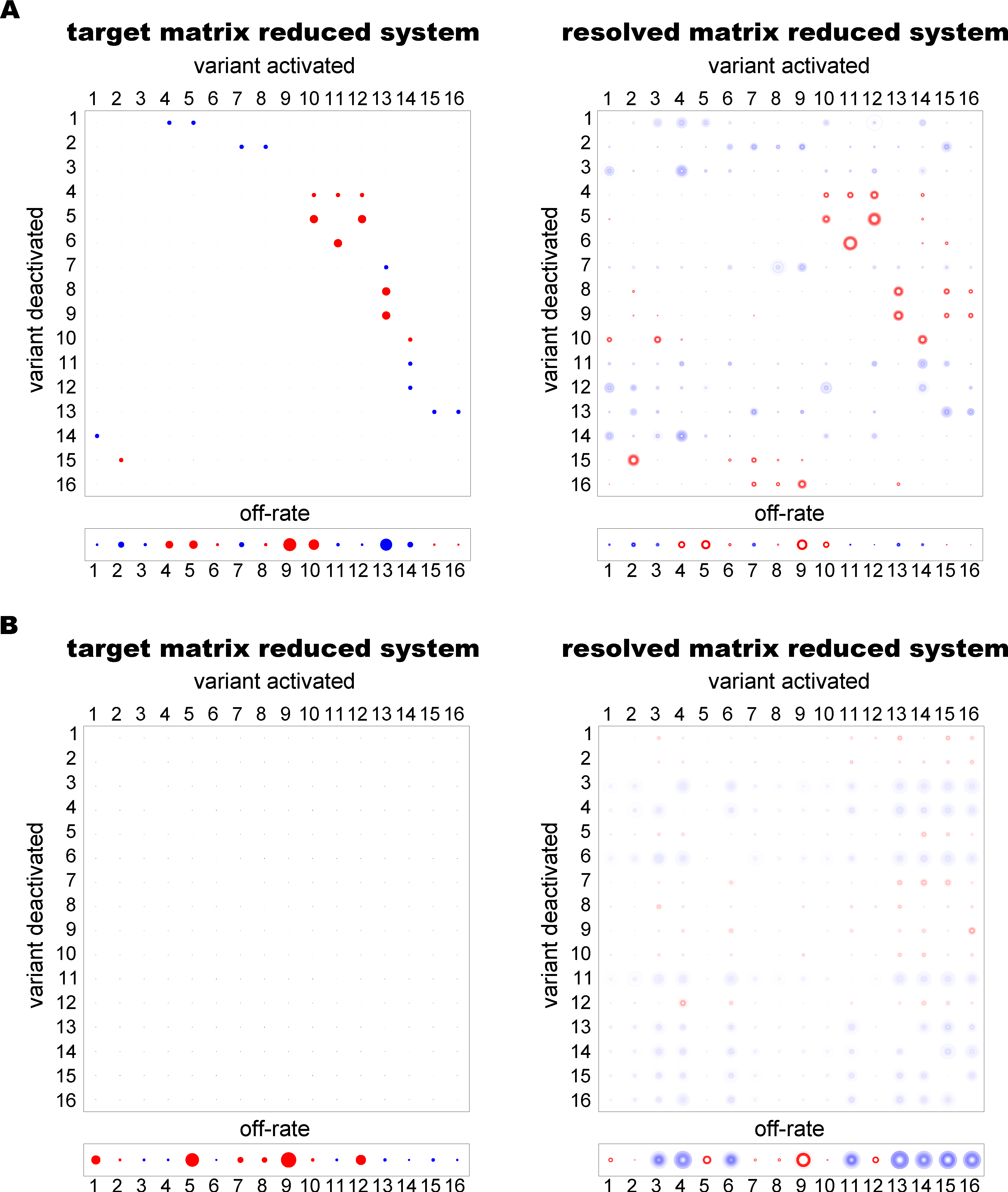

Supplement: Figure S2 — Results following dimension reduction. Left column: target parameters for the (A) lattice and (B) uniform networks reduced from 60 to 16 genes. Right column: MCMC output after adding noise with . To perform the reduction, genes were ranked by their average transcription levels across all time points and all cultures in the data generated by the 60-dimensional matrix (after adding noise). The 16 most highly ranked genes were then selected and their data renormalised. The starter gene parameters are shown in red. (TIFF) [file pone.0039335.s002.tiff]

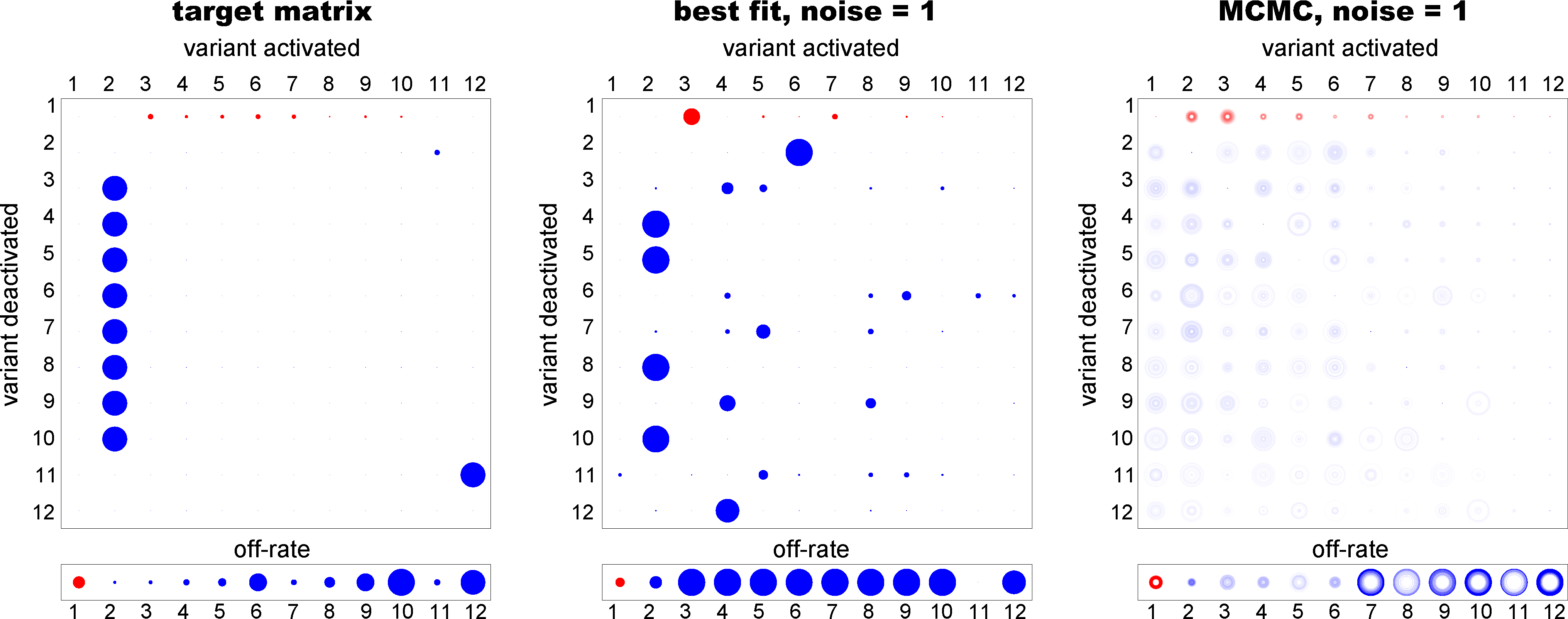

Supplement: Figure S3 — Results from simulating hypothesised var gene system. In this case the 60-gene SMS network was reduced to 12 genes by selecting only one starter gene, instead of the eight starter genes used in Figure 7. Simulated transcription levels were recorded at only three time points and a high level of noise was added to the data. These conditions mimic those of the experimental data sets IT4_2B2 (three time points recorded) and IT4_2F6 (four time points) analysed by Recker et al. [15] and the results are similar to those found by analysing the original data (Figure 8). The best-fit parameter estimates suggest an SMS network but the MCMC output shows that this result is uncertain because of the relatively small data set. (TIFF) [file pone.0039335.s003.tiff]
